# Clinical, Histopathological, and Prognostic Characteristics of Patients with Prostate Cancer in Lubumbashi, Democratic Republic of Congo

**DOI:** 10.1155/2020/5286929

**Published:** 2020-12-09

**Authors:** Pitchou Mukaz Mbey, Olivier Mukuku, Willy Kalau Arung, Guylain Kitoko Tengu, Nasser Lubosha Amisi, Véronique Kabila Kyabu, Etienne Fwamba Koshe Odimba, François Katombe Tshilombo

**Affiliations:** ^1^Department of Surgery, Faculty of Medicine, University of Lubumbashi, Lubumbashi, Democratic Republic of the Congo; ^2^Institut Supérieur des Techniques Médicales, Lubumbashi, Democratic Republic of the Congo; ^3^Centre Médical Baraka, Lubumbashi, Democratic Republic of the Congo; ^4^Hôpital Militaire de Garnison de Lubumbashi, Lubumbashi, Democratic Republic of the Congo; ^5^Department of Histopathology, Faculty of Medicine, University of Lubumbashi, Lubumbashi, Democratic Republic of the Congo

## Abstract

**Introduction:**

Prostate cancer is currently a public health problem with a frequency that varies from country to country. This study aims to describe the epidemiological, clinical, and histopathological and outcome features of prostate cancer in Lubumbashi in the Democratic Republic of Congo.

**Materials and Methods:**

This was a descriptive longitudinal study of patients diagnosed with prostate cancer at the University Clinics of Lubumbashi. The study period was 3 years (2017 to 2019). Parameters studied were age and clinical, biological (PSA level, prostatic specific antigen), histopathological, and outcome features.

**Results:**

The mean age of patients was 68.7 years (range: 47 and 90 years). The 60 to 69 age group was the most affected (43.18%). Elderly subjects (≥60 years old) represented 89.77% of the cases (*n* = 79). Voiding disorders were the main reason for consultation in 55.68% of the cases. The mean PSA level was 133.7 ng/ml (range: 4 and 1564.5 ng/ml) at diagnosis and 125.4 ng/ml after 3 months of follow-up (range: 0.16 and 1782.1 ng/ml). Adenocarcinoma was the predominant histological type (100%). In prognosis, 31.82% of patients had a Gleason score greater than 7 and 59.10% had a high risk at the D'Amico risk classification for Prostate Cancer. Hormone therapy was administered alone in 75% of the cases and in combination with pulpectomy in 13.64% of the cases. The 3-year overall survival was 56.82%.

**Conclusion:**

Prostate cancer is frequent and has a poor outcome in our country. The establishment of an individual screening policy would be an undeniable advantage in improving the prognosis.

## 1. Introduction

Prostate cancer (PCa) is one of the most common cancers in men [1]. In developed countries, it ranks second after lung cancer but ranks first with liver cancer in West Africa [2]. Globally, black men are more affected than whites. Its frequency varies according to the age pyramid, economic development, and access to healthcare for populations. It is estimated that by 2030 the global annual incidence will reach 1.7 million new cases [3]. Admittedly, this incidence has been increasing steadily since the popularization of diagnostic means, in particular by suspicious digital rectal examination and the determination of the prostate specific antigen (PSA) [4]. In the Democratic Republic of Congo (DRC), in Kinshasa, a screening study carried out on 162 men aged 40 to 70 years reported a detection rate of PCa of 2.5% [5]. However, it is difficult for us, as in most developing countries, to assess the extent and impact of this cancer due to the lack of epidemiological indicators.

This study aims to describe the epidemiological, clinical, and histopathological and outcome features of prostate cancer in Lubumbashi in the DRC.

## 2. Materials and Methods

### 2.1. Sampling and Patients

This was a longitudinal prospective study, conducted in the urology service of the University Clinics of Lubumbashi for a period of 3 years, from January 1^st^, 2017 to December 31^st^, 2019. The samples consisted of biopsies and operating pieces fixed in 10% formalin. These samples were processed using conventional histology techniques. Parameters studied were age at diagnosis, circumstances of discovery, PSA level at diagnosis and 3 months after initiation of treatment, histological analysis, Gleason score, D'Amico risk classification for PCa, treatment administered, and outcome of the patients.

The PSA assay was carried out using a HumanReader HS brand device with a PSA kit from the Human Diagnostics Worldwide laboratory (65205 Wiesbaden, Germany). For the prostate biopsy, we used sterile Fast-Cut disposable needles 20 cm long and 20 mm in diameter from the firm Sterylab (Milan, Italy). The biopsy was echo-guided using a Digital Color Doppler Ultrasound System model S11 (Sonomed, 74-00128 Rome, Italy) with an endorectal probe equipped with a device for the biopsy needle.

Per patient, twelve sextant biopsies (due to 3 cores in the 4 anatomical zones of the prostate described by Mc Neal) were fixed in 10% formalin in vials previously labeled, packaged, and shipped for histological analysis.

### 2.2. Data Collection and Analysis

Data collected were double cross-checked and edited for every existing error. Statistical analyses were performed using STATA version 12.0. Descriptive analysis was carried out using calculations of proportions for qualitative variables (frequency, percentage) and means (standard deviation [SD]) for the continuous variables. To compare frequencies, we used Pearson's chi-square test and the ANOVA test to compare means between different outcomes. Kaplan–Meier curves were used to analyze the 3-year overall survival rate of the cases. All cases without the event of interest (death) at the end of the follow-up period were censored. We considered as significance level a probability of error lower than 0.05.

### 2.3. Ethical Approval

Permission was obtained from the medical ethic committee of the University of Lubumbashi.

## 3. Results

### 3.1. Age

The age of the patients varied between 47 and 90 years with a mean age of 68.7 years; 79.5% of the patients were between 60 and 79 years old (Table 1).

### 3.2. Circumstances of Discovery

The telltale signs of PCa were dominated by voiding disorders presented by 49 patients representing 55.68% of the cases. The rest of the patients consulted for low back pain (10.23%) or hematuria (3.68%) or hemospermia (2.27%). The diagnosis of PCa was incidentally discovered during a health check in 32 patients (36.36%). In 44.32% of the patients, the digital rectal exam was malignant.

### 3.3. PSA Level

The mean PSA level at diagnosis was 133.7 ng/ml (range: 4 and 1564.5 ng/ml) and at 3 months of follow-up was 125.4 ng/ml (range: 0.16 and 1782.1 ng/ml).  Table 2 shows distribution of the patients according to the PSA level at diagnosis and at 3 months of follow-up.

### 3.4. Histological Features

Histologically, PCa was essentially adenocarcinoma observed in all of the cases in our series. Distribution of the patients, according to the Gleason score and the D'Amico risk classification, is presented in Table 2.

**Figure 1:**
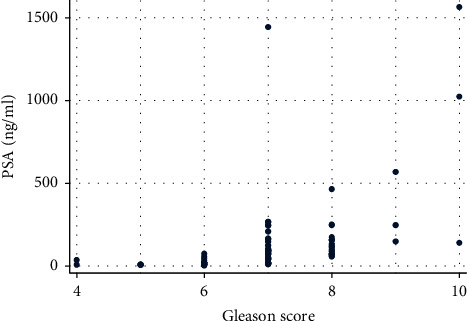
Correlation of PSA level at diagnosis as a function of the Gleason score.

PSA levels increase steadily based on the Gleason score. The analysis of the correlation between PSA level at diagnosis and Gleason score is illustrated in Figure 1.

### 3.5. Treatment

Regarding the treatment administered to our patients, 75% of the cases were put on hormone therapy alone and 13.64% in combination with a pulpectomy (Table 2).

### 3.6. Outcomes

After 3 years of follow-up, we recorded 24 deaths (27.27%) and 14 lost to follow-up (15.91%). The main causes of death in our patients were severe anemia (16/24), and chronic renal failure (11/24). The 3-year overall survival rate was 56.82%. The mean follow-up time for deceased patients was 12.33 months (range: 5 and 24 months).

Table 3 shows characteristics of the patients according to their outcome. We find that there is a statistically significant correlation between the appearance of PCa on digital rectal examination and outcome of the patients (*p*=0.0013); the same is true for mean PSA level and patients' outcome (*p* < 0.05). The mean PSA levels at diagnosis were 80.38 ng/ml, 268.35 ng/ml, and 93.53 ng/ml, respectively, in surviving, deceased, and lost to follow-up patients. The comparison of these means shows a statistically significant difference (*p*=0.007). At 3 months of follow-up, the mean PSA levels were 28.44 ng/ml, 357.66 ng/ml, and 73.39 ng/ml, respectively, in surviving, deceased, and lost to follow-up patients. These three means were statistically different (*p* < 0.0001). We found that means PSA level of deceased patients were significantly high compared to other patients.

**Figure 2:**
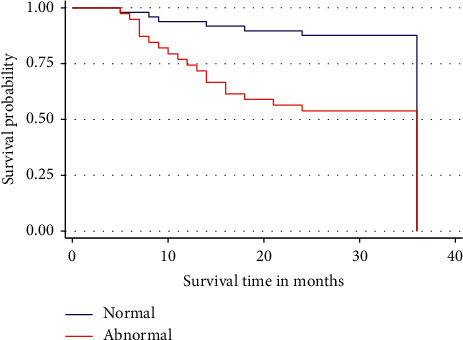
Three-year overall survival by prostate state on digital rectal examination of the patients. Patients with abnormalities show a relatively increased risk of dying compared to those without abnormalities.

The risk of dying among the patients included in this study was increasing with clinical stage. Patients without abnormalities on digital rectal examination were dying at a long interval compared to those with abnormalities (Figure 2). Additionally, it was also observed that the difference in the 3-year overall survival rate between the two groups was statistically significant (*p*=0.0013).

**Figure 3:**
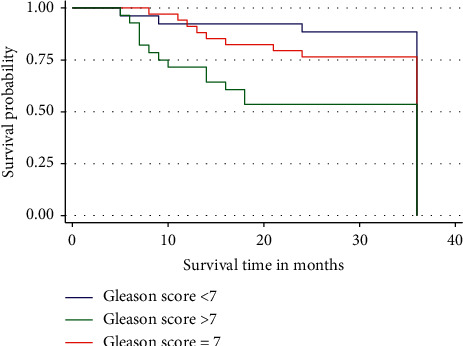
Three-year overall survival by Gleason score group of the patients. Patients with Gleason score >7 show a relatively increased risk of dying compared to those with Gleason score ≤7.

In this study, despite the risk of dying among patients with Gleason score >7, the 3-year overall survival rate was not significantly different from the patients who had Gleason score ≤7 (*p*=0.0513) although the ones with Gleason score were more likely to die than those with Gleason score ≤7. Even the Kaplan–Meier curves for the three Gleason score groups compared were almost overlying (Figure 3).

**Figure 4:**
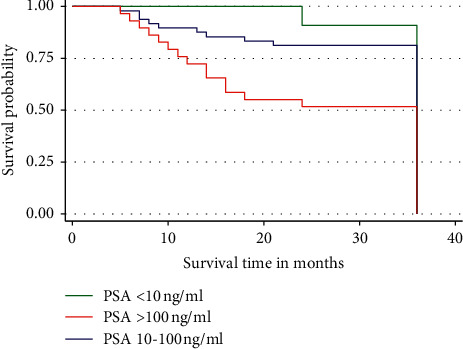
Three-year overall survival by PSA (at diagnosis) group of the patients. Patients with PSA >100 ng/ml show a relatively increased risk of dying compared to those with PSA ≤100 ng/ml.

The probability of dying among patients with PSA >100 ng/ml in our study was higher than those with PSA <10 ng/ml and PSA between 10 and 100 ng/ml. This contributed to the gap in the 3-year overall survival rate between the two groups although the survival rate was significantly different (*p* < 0.05) (Figure 4).

**Figure 5:**
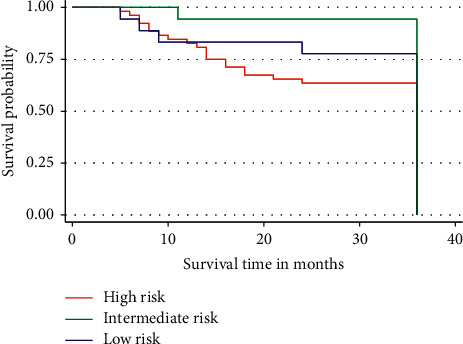
Three-year survival by D'Amico risk classification group of the patients. Patients with high risk show a relatively increased risk of dying compared to those with low and intermediate risks.

Figure 5 shows the Kaplan–Meier curves for D'Amico risk classification in relation to 3-year overall survival rate. The correlation of D'Amico risk classification and the 3-year overall survival rate was not statistically significant (*p* > 0.05). Patients in the group with high risk were more likely to die than patients with low and intermediate risks. In less than 10 months of overall survival after diagnosis, patients in all groups had almost equal survival. Thereafter, the survival rate of those at high risk decreased compared to those with low and intermediate risks.

## 4. Discussion

This preliminary study cannot be considered as globalizing all the cases of PCa observed in Lubumbashi, because only the flow of patients seen in consultation and follow-up was taken into account in the urology service of the University Clinics of Lubumbashi, which is responsible for ensuring biopsy samples. Some patients consult in private centers or outside the country or even die without consulting. Nevertheless, it translates the various current presentations of PCa in Lubumbashi.

This study finds 68.7 years as the mean age of the patients with PCa, comparable to those found in the African literature reporting a mean age of 69 years [4, 6, 7]. Other African and European studies have noted a mean age of 71 years [8, 9] reflecting that PCa is a disease of the elderly. According to Villiers and Grosclaude [10], it is the most common cancer in men over 50 years of age. In this study, 2 (2.27%) patients were exceptionally less than 50 years old with the notion of cancer in first degree relatives. However, it should be noted that this notion of family cancer in our series had not been documented and was based only on patient claims. The mean age at diagnosis was high, hence the presence of these very advanced forms beyond any therapeutic resource, especially in our context. The delay in diagnosis is linked to the natural history of PCa but also to the apprehensions that men had coming to see the urologist. In addition, there are a lack of information and awareness-raising policy among the population on this condition and difficulties in accessing PCa screening services [11].

Clinically, voiding disorders (55.68%) and spinal bone pain (10.23%) were frequent reasons for consultation. Botto et al. [12] found 69% and 70%, respectively, for voiding disorders and spinal bone pain; Magoha [13] also noted 64.5% for spinal pain. The existence of functional manifestations is a constant witness to the spread of cancer pathology. Voiding disorders frequently revealed PCa and this prevalence is reported in the literature [4].

With the advent of Pca screening, incidental discoveries just about elevating PSA levels without clinical signs become frequent [14]. In the present study, we also observed 36.36% of incidental discovery during a health check, the result of awareness for individual screening. But this rate is still very low compared to European studies because this individual screening is not yet common practice in our country.

On digital rectal examination, abnormalities in favor of Pca were observed in 44.32% of our patients. Tengue et al. [15] reported 81.9% of cases with abnormalities on the digital rectal examination. This is mainly due to an advanced stage of Pca as is the case in most African series [7, 13, 15]. This is also demonstrated in the present study where we found that there was a statistically significant correlation between the prostate abnormality on digital rectal examination and mortality (*p*=0.0013).

In the present study, mean PSA level is relatively high (133.7 ng/ml). Mean values vary according to studies: 120 ng/ml for Diallo et al. [16] in Guinea, 121.94 ng/ml for Troh et al. [17] in Côte d'Ivoire, 125.7 ng/ml for Niang et al. [18] in Senegal, and 419.71 ng/ml for Engbang et al. [19] in Cameroon. This situation could be explained by an often-late diagnosis of PCa in our country. However, these values are not always a function of the degree of tumor differentiation, which shows great variability in PSA levels compared to the histological type. PSA (biological reference marker) is a kallikrein primarily secreted by prostate epithelial cells under the control of an androgen-dependent gene. The interest of its dosage in prostatic pathology (cancerous or benign) is well known: diagnostic aid, prognostic value, monitoring of the effectiveness of treatment, and detection of recurrences and metastases [18, 20, 21]. In the present study, we found that the mean PSA levels (at diagnosis and at 3 months of follow-up) were statistically very high in the deceased patients compared to those who survived, reflecting a strong association between the PSA level and patients' outcome.

In terms of diagnosis, only conventional histopathology techniques are practiced in Lubumbashi where histopathological examination remains the only means of diagnosing PCa. The absence of labeling techniques makes diagnosis sometimes difficult. In our study, adenocarcinomas represent most of the histological types as in the literature [17, 22–24]. According to Mostofi et al. [25], other types are rare (around 3%) and their diagnosis requires the contribution of different techniques not accessible in our environment outside the standard PSA dosage.

Therapeutically, the majority of our patients had received first-line hormone therapy in 75% of the cases as in the series by Tengue et al. [15] in Togo, sometimes associated with pulpectomy in 13.64% of the cases. This indicates the advanced stage of these tumors. Patients with locally advanced cancer had received only hormone therapy while hormone radiotherapy is recommended [26], a method which is not available in Lubumbashi due to lack of adequate technical platform in our structures. The initiation of treatment, particularly hormone therapy, leads to a considerable reduction in clinical manifestations [27]. Similarly, PSA drops also at a variable rate in three to six months, and the lowest rate observed has a prognostic value on survival without recurrence [15].

In this study, we observed at three months of follow-up a decrease in the PSA level in surviving patients (low risk cancer) with a mean PSA level of 28.44 ng/ml (range: 6.5 and 89.32 ng/ml), while in deceased patients the mean PSA level was 357.66 ng/ml (range: 88.13 and 1782.10 ng/ml). This supposes the existence of aggressive forms in the deceased patients faced with soaring PSA levels instead of its decrease. Only 4 patients benefited from a radical prostatectomy performed by retropubic route, a technique which deserves to be popularized more and which does not yet have an easy adhesion on the part of our patients.

The present study shows that the 3-year survival rate was 56.82%. Other authors have found 1-year survival rates of 86% [28] and 91% [15] after hormone therapy. Some parameters have been associated with a significant decrease in overall survival: high PSA levels and prostate abnormalities on digital rectal examination. This observation reflects the spread of PCa from a distance in most of our patients. A Ugandan study reported a 3-year overall survival rate of 67.6% with a median survival of 36.5 months [29]. These authors had found that Gleason score, clinical stage, and lymphovascular invasion can independently predict the overall survival of the patients with PCa [29]. Ndoye et al. [8] emphasize not only the delay in diagnosis observed in most sub-Saharan countries but also the lack of policy of channeling these patients suffering from PCa to a center applying the recommendations of learned societies for a codification of management.

The present study reports a poor prognosis in patients followed for prostate cancer. Hence the introduction of this screening in our context could improve the prognosis of our patients while limiting itself to an individual PSA screening after having provided the adapted and informed information to the men wishing a PSA assay on the advantages and disadvantages of the screening, and in particular the risks associated with the diagnostic process and the treatment. A European study demonstrated a reduction in specific mortality linked to prostate cancer of around 20% (relative risk (RR): 0.79; 0.69-0.91) in the group of patients who received PSA screening [30].

One of the contributing factors is the ecological peculiarities of the south-eastern region of the DRC, in particular the province of Katanga where the intensification of metallurgical activities of extraction and processing of ores expose men to physicochemical conditions different from those of the rock putting in the environment potentially toxic trace elements and heavy metals whose effects on the prostate may explain the aggressive forms of prostate cancer observed. In the study by Saleh et al. [31], an association has been observed between disturbance of serum trace elements and prostate cancer. Reduced levels of Se, Zn, and Mn and increased levels of Cu and Fe may play an important role in the initiation of prostate cancer. However, future prospective studies are needed to illustrate the relationship between the different stages of prostate cancer and the levels of trace elements.

## 5. Conclusion

PCa remains a reality in our environment, its diagnosis often occurs at an advanced stage of the disease, and its clinic features are dominated by voiding disorders and spinal pain. Efforts must be made to improve individual screening because the earlier the diagnosis, the better the management strategy to improve survival.

## Figures and Tables

**Table 1:** Demographic and clinical characteristics of the patients.

Variable	Number (*n* = 88)	Percentage
*Age*		
<50 years	2	2.27
50–59 years	7	7.95
60–69 years	38	43.18
70–79 years	32	36.36
≥80 years	9	10.23
Mean ± SD (range)	68.73 ± 8.02	47–90

*Comorbidity*		
None	61	69.32
High blood pressure	15	17.05
Diabetes	8	9.09
Respiratory failure	3	3.41
Heart failure	2	2.27

*Circumstances of discovery*		
Voiding disorder	49	55.68
Lucky find	32	36.36
Spinal pain	9	10.23
Edema of the lower limbs	5	5.68
Hematuria	3	3.41
Hemospermia	2	2.27
Paraplegia	2	2.27

*Prostate state on digital rectal examination*		
Normal	49	55.68
Abnormal	39	44.32

**Table 2:** PSA level, prognostic features, and different treatment approaches of the patients.

Variable	Number (*n* = 88)	Percentage
*PSA level at diagnosis*		
<10 ng/ml	11	12.50
10–100 ng/ml	48	54.55
>100 ng/ml	29	32.95
Mean (range)	133.7	(4–1564.5)

*PSA level at 3 months of follow-up*		
<4 ng/ml	17	19.32
4–9.9 ng/ml	10	11.36
10–100 ng/ml	30	34.09
>100 ng/ml	31	35.23
Mean (range)	125.4	(0.16–1782.1)

*Gleason score*		
<7	26	29.55
7	34	38.64
>7	28	31.82

*D'Amico risk classification*		
Low risk	18	20.45
Intermediate risk	18	20.45
High risk	52	59.10

*Treatment*		
Hormone therapy	66	75.00
Hormone therapy + pulpectomy	12	13.64
Surveillance	6	6.82
Radical prostatectomy	4	4.55

*Outcome*		
Survived	50	56.82
Deceased	24	27.27
Lost to follow-up	14	15.91

**Table 3:** Characteristics of the patients according to their outcomes.

Variable	Survived (*n* = 50)	Deceased (*n* = 24)	Lost to follow-up (*n* = 14)	*p* value
*Age (in years)*				
Mean (range)	68.48 (49–90)	68.79 (47–83)	69.57 (60–80)	0.905

*PSA level at diagnosis (in ng/ml)*				
Mean (range)	80.38 (6.5–89.32)	268.35 (9–1564.50)	93.53 (4–251.00)	0.007

*PSA level at 3 months of follow-up (in ng/ml)*				
Mean (range)	28.44 (0.16–87.12)	357.66 (88.13–1782.10)	73.39 (0.23–201.16)	<0.0001

*Comorbidity*				0.6966
Present	14 (28.00%)	9 (37.50%)	4 (28.57%)	
Absent	36 (72.00%)	15 (62.50%)	10 (71.43%)	

*Prostate on digital rectal examination*				0.0013
Normal	35 (70.00%)	6 (25.00%)	8 (57.14%)	
Abnormal	15 (30.00%)	18 (75.00%)	6 (42.86%)	

*D'Amico risk classification*				0.1163
Low risk	12 (24.00%)	4 (16.67%)	2 (14.29%)	
Intermediate risk	13 (26.00%)	1 (4.17%)	4 (28.57%)	
High risk	25 (50.00%)	19 (79.17%)	8 (57.14%)	

*Gleason score*				0.0513
<7	19 (38.00%)	3 (12.50%)	4 (28.57%)	
7	19 (38.00%)	8 (33.33%)	7 (50.00%)	
>7	12 (24.00%)	13 (54.17%)	3 (21.43%)	

## Data Availability

The datasheet used to support the findings of this study are available from the corresponding author upon request.
